# Risk‐reducing mastectomy decisions among women with mutations in high‐ and moderate‐ penetrance breast cancer susceptibility genes

**DOI:** 10.1002/mgg3.2031

**Published:** 2022-08-25

**Authors:** Jacob G. Comeaux, Julie O. Culver, John E. Lee, Danielle Dondanville, Heather L. McArthur, Emily Quinn, Nicholas Gorman, Charité Ricker, Ming Li, Caryn Lerman

**Affiliations:** ^1^ Norris Comprehensive Cancer Center University of Southern California Los Angeles California USA; ^2^ Samuel Oschin Cancer Center Cedars‐Sinai Medical Center Los Angeles California USA; ^3^ Department of Internal Medicine University of Texas Southwestern Medical Center Dallas Texas USA; ^4^ Human Genetics and Genomics Keck Graduate Institute Claremont California USA

**Keywords:** *BRCA1*/2, breast cancer, decision‐making, mastectomy

## Abstract

**Background:**

Women harboring mutations in breast cancer susceptibility genes are at increased lifetime risk of developing breast cancer and are faced with decisions about risk management, including whether to undergo high‐risk screening or risk‐reducing mastectomy (RRM). National guidelines recommend *BRCA1* or *BRCA2* mutation carriers consider RRM, but that carriers of moderate penetrance mutations (e.g., *ATM* or *CHEK2*) should be managed based on family history. We aimed to investigate determinants of decision for RRM, and hypothesized that mutation status, age, family history, partner status, and breast cancer would impact RRM decision making.

**Methods:**

We performed a retrospective study assessing RRM decisions for 279 women.

**Results:**

Women with *BRCA* and moderate penetrance gene mutations, a personal history of breast cancer, or a first degree relative with a history of breast cancer were more likely to undergo RRM. Breast cancer status and age showed an interaction effect such that women with breast cancer were less likely to undergo RRM with increasing age.

**Conclusion:**

Although national guidelines do not recommend RRM for moderate penetrance carriers, the rates of RRM for this population approached those for *BRCA* mutation carriers. Further insights are needed to better support RRM decision‐making in this population.

## Graphical abstract

Surgical decisions varied between women with and without breast cancer and by mutation status. Our study showed higher rates of RRM among breast cancer patients than among unaffected patients and higher rates of RRM by mutation risk level in both affected and unaffected patients.

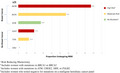

## 1 INTRODUCTION

While decision making for risk management has been studied for *BRCA* mutation carriers, there is a paucity of information to inform risk management decision‐making for women with moderate penetrance gene mutations such as mutations in *ATM* (OMIM accession number: 607585) or *CHEK2* (OMIM accession number: 604373). The genes *BRCA1* (OMIM accession number: 113705) and *BRCA2* (OMIM accession number: 600185) were discovered in the mid‐1990s and are considered “high penetrance” because mutations confer, by some estimates, a greater than 80% lifetime risk for female breast cancer (Easton, 1999, 2015; Kapoor, 2015; Kuchenbaecker, 2017). Mutations in these two genes are thought to account for approximately half of all hereditary breast cancers (Tedaldi, 2017). The successful development and implementation of next‐generation DNA sequencing permitted simultaneous identification of multiple newly discovered gene mutations that were subsequently implicated in hereditary cancers (Powers, 2018). These genes include “moderate penetrance” breast cancer susceptibility genes such as *ATM* and *CHEK2*, mutations in which are associated with a 2‐4‐fold increase in lifetime risk of female breast cancer (Antoniou, 2014; Easton, 2015; LaDuca, 2020; Leedom, 2016; Marabelli, 2016).

In general, *BRCA* carriers begin breast awareness at age 18, initiate clinical breast examination every six to twelve months at age 25, commence radiographic screening beginning at age 25 until at least age 75 with age‐dependent frequencies and modalities including annual breast MRI and mammogram, are counseled about the option of risk‐reducing mastectomy (RRM), and are advised to undergo risk‐reducing oophorectomy after completion of childbearing (Domchek, 2006; Jatoi, 2016; Jernström, 2004; Kotsopoulos, 2018; NCCN Clinical Practice Guidelines, 2019, 2020). During the time frame of the study, for mutation carriers in *PALB2*, *ATM*, *CHEK2*, and *NBN* (OMIM: 602667) carriers, the NCCN Guidelines® (v.3.2019) recommended annual mammogram and consideration of breast MRI starting at age 40 but cautioned that evidence was insufficient for recommending RRM which should be “based on family history” (NCCN Clinical Practice Guidelines, 2019). Women with newly diagnosed breast cancer are increasingly offered genetic panels to explore whether they are at an increased risk for developing future breast cancers so that they can make informed decisions regarding surgical approach (Kurian, 2018; Weitzel, 2021).

Risk‐reducing mastectomy is the most risk‐conservative but also invasive procedure indicated for women at a high risk of breast cancer, which reduces breast cancer risk in unaffected *BRCA* carriers (RR = 0.05–0.114) (Honold, 2018; Li, 2016). Affected *BRCA* mutation carriers who undergo contralateral RRM also decrease the risk of contralateral breast cancer risk significantly (RR = 0.072) (Li, 2016). However, research is conflicting as to whether RRM is significantly associated with reduced mortality for *BRCA* carriers (Honold, 2018; Li, 2016). Uptake of RRM for all breast cancer patients, regardless of mutation status, has increased due to the overestimation of both risks of contralateral breast cancer (CBC) and degree of risk reduction obtained by RRM (Ager, 2016; Chiesa, 2016; Fagerlin, 2006; Jagsi, 2017). Furthermore, while young age at first primary breast cancer diagnosis confers a higher risk for CBC, it is unclear the degree of risk reduction or the survival benefit from contralateral RRM in young women with early‐stage breast cancer (Teoh, 2020). Decision‐making about RRM requires comprehension of both the efficacy of the procedure and the adverse events and risks involved, but research suggests that the decision‐making process is often driven by subjective concerns rather than risk–benefit calculations for many women (Katz, 2007; Rosenberg, 2013; Scott, 2003; Zikmund‐Fisher, 2010).

Most data regarding RRM decision‐making for gene positive women without a personal history of breast cancer pertains to *BRCA* mutation carriers. Country of origin, timing of genetic testing, screening discomfort, cancer worry, family history, and the “Angelina Effect” observed after the public announcement of bilateral RRM by the actress Angelina Jolie, are suggested to impact surgical decision for unaffected *BRCA* mutation carriers (Evans, 2009, 2019; Henry, 2019; Hoskins, 2012; Liede, 2018; Metcalfe, 2019; Skytte, 2010; Chalmers & Thomson, 1996). There have been, however, recent contributions to the behavior of women with moderate penetrance mutations. One study including 21 *ATM* and *CHEK2* carriers demonstrated that 52% of them elected RRM, clearly underscoring the need for further research into this topic, especially given the lack of clinical guidelines supporting RRM in this population (Cragun, 2020). Another study explored surgical decisions among 16 women with mutations in moderate penetrance breast cancer genes using semi‐structured qualitative interviews, including 4 unaffected with breast cancer. Decision‐making factors emerging from semi‐structured interviews included family history, physician opinions, risk perception, sibling influence, and health insurance (Napoli, 2020).

Our study aimed to identify the impact of mutation status, family history, age, partner status, and personal breast cancer status with the decision to undergo RRM among both affected and unaffected women undergoing genetic testing. We hypothesized that women were more likely to undergo RRM if they were younger, affected with breast cancer, partnered, had a first degree relative with breast cancer, or had a high‐penetrance mutation. Results of this analysis will inform future research efforts to identify tailored approaches to patient education and decision‐making support.

## 2 MATERIALS AND METHODS

### 2.1 Ethical compliance

This study was approved by the Cedars‐Sinai Institutional Review Board.

### 2.2 Eligibility and patients

An institutional database was used to identify women with genetic test results reported 6/1/2009 through 6/1/2019. Eligible women were at least 18 years of age with an available pedigree or family history, a documented management plan and surgical decision, documented mutation status before final surgical decision making, and had undergone counseling with Cancer Genetics at Cedars‐Sinai Medical Center in Los Angeles, CA. Both breast cancer patients and women unaffected with breast cancer were included in this study population.

Patients with prior breast surgery before receiving genetic testing results, an unknown family history, stage 4 breast cancer, being actively treated for other cancers, or with breast cancer gene mutations other than those defining their study group were ineligible.

Gene mutation group status was defined as carrying a mutation (pathogenic or likely pathogenic variant) in a moderate penetrance gene (*ATM* (NM_000051.4), *CHEK2* (NM_001005735.2), *NBN* (NM_001024688.3), or *PALB2* (NM_024675.4)), high penetrance gene (*BRCA1* (NM_007294.4) or *BRCA2* (NM_000059.4)), or having no known mutation identified on hereditary cancer panel testing.

In order to populate our moderate penetrance group a list of genetic test results in the defined time period was filtered to include mutations in *ATM*, *CHEK2*, *NBN*, and *PALB2*. *PALB2* and *NBN* were listed as a moderate penetrance breast cancer gene during this study's eligibility window but, as expanded upon in the limitations section of this paper, recent publications have estimated *PALB2* to confer a higher risk for breast cancer than previously thought. Since this study was conducted, *NBN* has been challenged in its association with breast cancer risk. For the purposes of our study, both of these genes were included as a moderate penetrance gene due to its classification and NCCN guidelines recommendations at the time of our study. We performed medical chart review of each patient, and 90 of 203 moderate gene mutation carriers met eligibility criteria for this study after medical chart review. The high risk *BRCA* mutation group was also populated using the same patient list, yielding a list of 300 potentially eligible subjects. Of the group of 300 *BRCA* carriers there were 30 eligible subjects diagnosed with breast cancer and 68 eligible unaffected subjects. As genetic testing started to be performed before surgical decision‐making through the 2010's, results became more often available to guide surgical decision and therefore there were more eligible subjects in more recent years. This practice coincided with larger multi‐gene panels becoming available which routinely included the moderate penetrance genes. Given that, the proportion of affected, eligible *BRCA* carriers (10%) was somewhat lower than the proportion of affected, eligible moderate penetrance gene carriers (17%). The “no mutation” status arm group was populated using a list of 813 patients who tested negative on genetic testing. A random integer generator was used to select a comparator group in a systematic random selection until 30 eligible subjects with breast cancer and 62 eligible women without breast cancer were accrued.

### 2.3 Study procedure

We performed a retrospective review of electronic medical records. Demographics, cancer history, mutation status, breast cancer risk, detailed personal history, three‐generation family history, surgical decision, and previous genetic testing were abstracted from each patient. Data on each patient was collected at the time of her genetic test result disclosure. Age at surgical decision was defined as the age at genetic test results.

### 2.4 Statistical analysis

Following descriptive analysis of the study population, we performed univariate analysis to examine the relationship between the surgical decision and each risk factor considered in this study, using Pearson's chi‐square test for categorical risk factors and Wilcoxon rank sum test for continuous risk factors.

Multivariable logistic regression modeling was performed to examine the joint effects of patient variables on the surgical decisions. Model selection by likelihood ratio test showed that a model including interaction effect between age and breast cancer was optimal to study the joint effect of age, family history (whether a first degree relative was affected with breast cancer), mutation status, partnered status and breast cancer status on RRM decision. After applying bootstrap cross validation, the bias‐corrected rank correlation between the predicted probabilities and the observed responses is 0.8025; the bias‐corrected c index (AUC) is 0.9013 (Table S1).

Log‐likelihood test was used for final model selection. The main and interaction effects were reported by odds ratios (ORs) and 95% confident intervals (95% CIs). Interaction plot was also provided to assist a better understanding of the effects. The predictive performance of the final model was assessed by the bias‐corrected c‐index (AUC).

## 3 RESULTS

### 3.1 Patient demographics and univariate analysis

**FIGURE 1:**
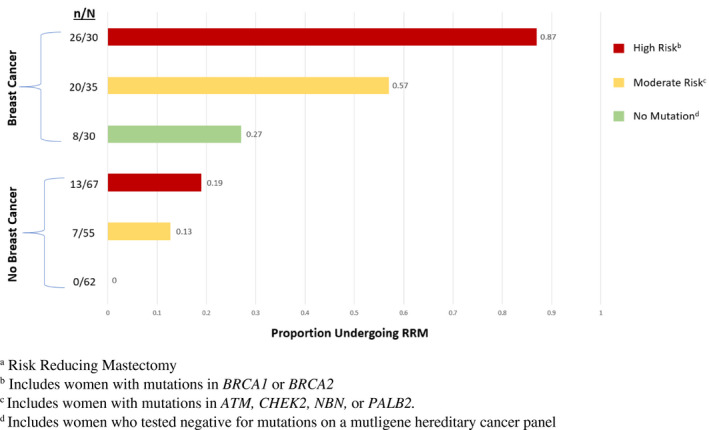
RRM rate by mutation and breast cancer status. Shown are the ratios of women electing for RRM separated by breast cancer status and stratified by risk group

We investigated RRM decisions for 279 women across three genetically defined risk groups. Our study included 23 *ATM*, 54 *CHEK2*, 3 *NBN*, 9 *PALB2*, 51 *BRCA1*, and 46 *BRCA2* carriers in addition to 92 patients who did not harbor a mutation. Results showed that 40.2% (39/97) of *BRCA* mutation carriers, 30% (27/90) of moderate penetrance gene carriers, and 8.6% (8/92) of women with no mutation underwent RRM. Patient demographics by RRM decision are listed in Table 1. Most of the patients included in this study were non‐Hispanic, White (78.1%). The average age at genetic test results was 45.7 years (22–90). Most patients diagnosed with breast cancer were diagnosed with invasive ductal carcinoma (70.5%) and most were either stage 1 or 2 breast cancer (77.9%). In multivariable logistic regression analysis, patients were more likely to undergo RRM if they had a personal history of breast cancer (*p* < .001), the younger they were diagnosed (*p* < .001), if they carried a high or moderate risk mutation (*p* < .001), or if they had a partner (*p* = .02) (Table 1). Surgical decisions varied between women with and without breast cancer and by mutation status, as shown in Figure 1, indicating higher rates of RRM among breast cancer patients than among unaffected patients, with higher rates by mutation risk level in both affected and unaffected patients.

**TABLE 1:** Risk reducing mastectomy by demographics

	No RRM^a^ *N* = 205	RRM *N* = 74	p*
**Characteristic**	**Mean (Range)**	**Mean (Range)**	
Age at genetic test results^b^	44 (19–90)	45 (21–67)	.71
**Characteristic**	**Mean (*SD*)**	**Mean (*SD*)**	
Age diagnosis (if applicable)	58.9 (12.9)	45.7 (9.3)	<.001
**Characteristic**	**Frequency (%)**	**Frequency (%)**	p*
Study population	205 (73.5)	74 (26.5)	
Family history of breast cancer			.787
Yes	142 (74)	50 (26)	
No	63 (72.4)	24 (27.6)	
First degree relative affected with breast cancer			.762
Yes	79 (72.5)	30 (27.5)	
No	126 (74.1)	44 (25.9)	
Second degree relative affected with breast cancer			.729
Yes	106 (72.6)	40 (27.4)	
No	99 (74.4)	34 (25.6)	
Race/Ethnicity			.87
Asian	14 (66.7)	7 (33.3)	
Black/African American	9 (75)	3 (25)	
White, Hispanic	6 (66.7)	3 (33.3)	
White, Non‐Hispanic Latino	57 (26.1)	161 (73.9)	
Other	15 (78.9)	4 (21.1)	
Partner status			.02
Partnered^c^	74 (84.1)	14 (15.9)	
Not partnered^d^	117 (68.8)	53 (31.2)	
Risk status			<.001
No known risk mutations	84 (91.3)	8 (8.7)	
Moderate risk (ATM, CHEK2, NBN, PALB2)	63 (70.0)	27 (30.0)	
High risk (BRCA1 or BRCA2)	58 (59.2)	39 (39.8)	
Diagnosed with breast cancer			<.001
Yes	41 (43.2)	54 (56.8)	
No	164 (89.1)	20 (10.9)	
Stage of breast cancer			.38
0	8 (53.3)	7 (46.7)	
1	17 (43.6)	22 (56.4)	
2	12 (34.3)	23 (65.7)	
3	4 (66.7)	2 (33.3)	

*Notes*: Genes studied include *ATM* (NM_000051.4), *CHEK2*_(NM 001005735.2), *NBN* (NM_001024688.3), *PALB2* (NM_024675.4), *BRCA1* (NM_007294.4), and *BRCA2* (NM_000059.4).

^a^ Risk reducing mastectomy.

^b^ Age at time of genetic test results coincided with age at surgical decision.

^c^ Includes married and unmarried couples.

^d^ Includes single never married women, widowed women, and divorced/separated women.

Among the 27 women with a moderate risk gene mutation who underwent RRM, 22 (81.5%) had a family history of breast cancer in a first, second, or third degree relative. In fact, 13 (48.1%) of the 27 women with a moderate risk mutation who underwent RRM had a first degree relative with a history of breast cancer. Each of the 7 women with moderate risk mutations with no personal history of breast cancer who underwent RRM had a first degree relative (FDR) with breast cancer (Table S2).

### 3.2 Multivariable Logistic Regression Analysis

A multivariable model was developed to determine whether age, breast cancer diagnosis, first degree relative with breast cancer, mutation, and partnered status impacted RRM decision.

Odds ratios for this model are demonstrated in Table 2.

**TABLE 2:** Multivariable logistic regression analysis odds ratios

Factor	ORs	0.95 CI
Age at genetic test results (Breast Cancer = Yes)^a^	0.097	[0.029, 0.323]
Age at genetic test results (Breast Cancer = No)^a^	1.193	[0.554, 2.570]
Diagnosed with breast cancer—Yes: No	50.731	[18.375, 140.061]
First degree relative—Yes: No	3.340	[1.443, 7.733]
High risk: No mutation	20.899	[6.278, 69.570]
Moderate risk: No mutation	7.967	[2.574, 24.662]
High risk: Moderate risk	2.623	[1.082, 6.361]
Partnered: Not partnered	2.103	[0.906, 4.880]

^a^ Note that the ORs of RRM decision is calculated using Age at 75% quantile vs. Age at 25% quantile (to roughly represent older versus younger women) for Breast Cancer Status separately by taking into the Age and Breast Cancer status interaction effect.

**FIGURE 2:**
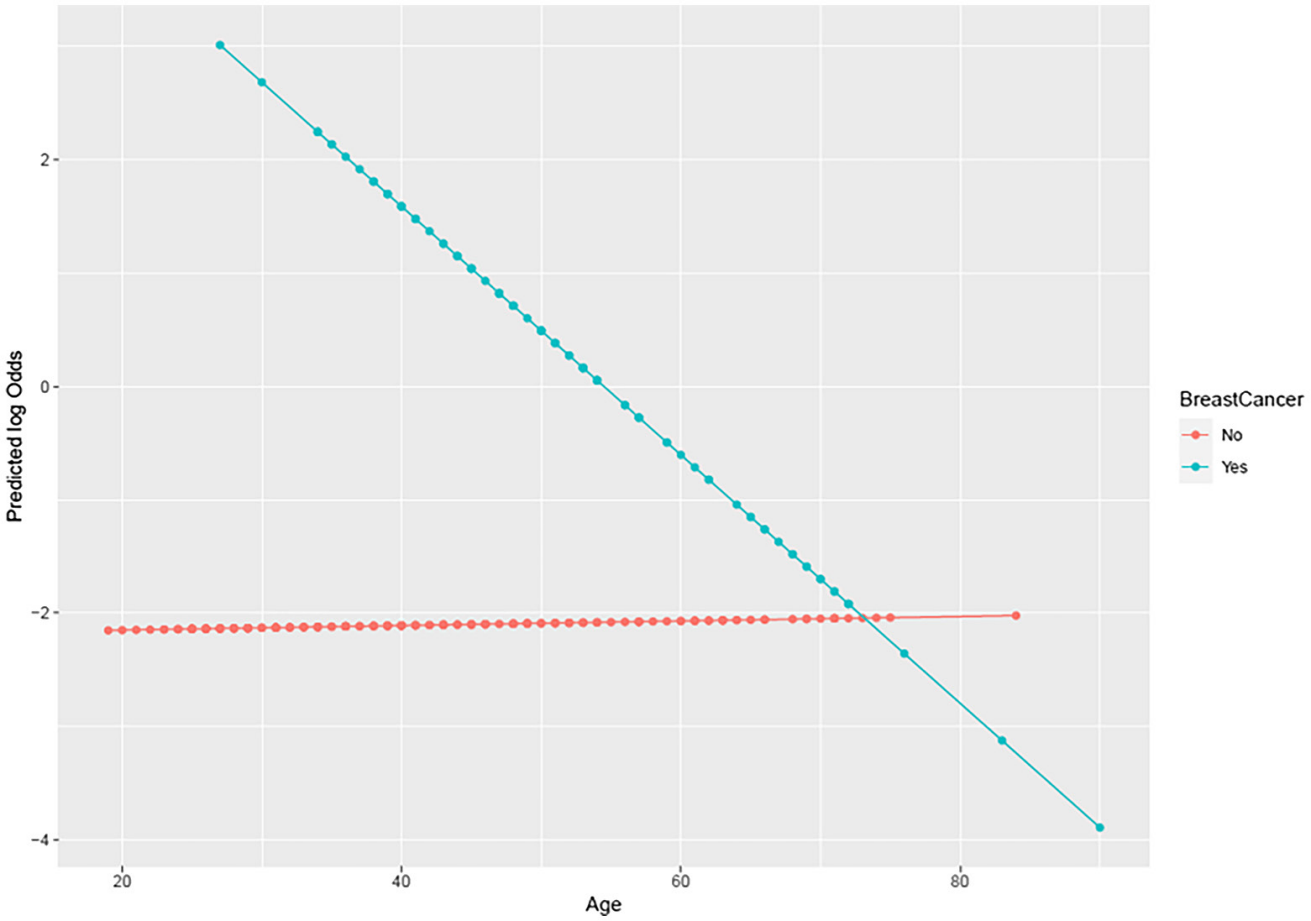
Predicted log odds interaction plot: Age × breast cancer. Shown is a simplified model demonstrating the predicted log odds of undergoing a RRM by age for women with breast cancer and women without breast cancer

Women with breast cancer were 50.731 times as likely to undergo a RRM as women without breast cancer, which was the strongest predictor of behavior [95% CI 18.375, 140.061]. Those with FDRs were over three times as likely to undergo RRM [95% CI 1.443, 7.733], and those with partners were more than two times as likely to undergo RRM, although the effect of having a partner was not statistically significant in the multivariable model [95% CI 0.906, 4.880]. Compared to women without mutations, the odds of undergoing RRM was of 20.899 [95% CI 6.278, 69.570] times higher for *BRCA* carriers and 7.967 [95% CI 2.574, 24.662] times higher for carriers of moderate penetrance genes. Women with *BRCA* mutations were 2.623 times as likely to undergo RRM as women with mutations in moderate penetrance genes [95% CI 1.082, 6.361]. Age was only a predictor of RRM among women with breast cancer. Among women with a diagnosis of breast cancer, the odds of undergoing RRM for older patients (75% quantile) compared to younger patients (25% quantile) was OR = 0.097 [95% CI 0.029, 0.323] (Table 2). Breast cancer status and age showed an interaction effect such that younger women with breast cancer were more likely to undergo RRM with increasing age, but age was not associated with RRM among women without breast cancer (Figure 2).

## 4 DISCUSSION

Though other researchers have studied the impact of multigene panel testing on surgical decision, this is the first study comparing RRM decision between high‐risk, moderate risk, and no mutation groups. Our study found that women with mutations in moderate penetrance genes are almost 8 times as likely to undergo RRM as women without mutations, a surprisingly high number given insufficient evidence to recommend this procedure. The NCCN Guidelines recommend that RRM decision for women with moderate penetrance mutations should be managed based on family history, but half of women who underwent RRM in this risk group did not have an affected first degree relative. It remains unclear how family history is guiding management in many cases. Across all genetically stratified groups in our study, women with a first‐degree relative with breast cancer were more than three times as likely to undergo RRM, suggesting that family history is a significant consideration in surgical decision making regardless of mutation status. Finally, in the entire study population, the odds of a woman with breast cancer undergoing RRM decreased with age, as demonstrated in a novel age and breast cancer interaction effect.

The identification of moderate penetrance gene mutations can be significant despite limitations of our knowledge regarding these genes. This is particularly important because patients' understanding of hereditary cancer panel testing may be less nuanced which provides unique psychosocial challenges. Culver et al. recently published a prospective study of 1264 participants assessing psychosocial outcomes following genetic testing and reported that participants with high or moderate risk mutations had higher levels of uncertainty, distress, and concerns about their testing experience than participants who tested negative or had variants of uncertain significance. While these researchers demonstrated that moderate risk mutation carriers experience their genetic test results similarly to those with high‐risk mutations, those with high‐risk mutations understood options for cancer screening and prevention better than the moderate risk group (Culver, 2021). In addition, evolving evidence supporting moderate risk genes may result in patients receiving conflicting interpretations of their results and estimated risks (Hamilton & Robson, 2019). Previous research by Cragun et al also demonstrated a possible overtreatment among women with mutations in *ATM*, *CHEK2*, and *PALB2*. Both studies found that mutation status, age, and breast cancer diagnosis are associated with RRM decision, but our study observed lower rate of RRM in unaffected women with a moderate risk of mutation. In their study 60% (12/20) of *PALB2* and 58% (7/12) of *ATM/CHEK2* carriers with breast cancer underwent RRM compared to 57% (4/7) and 29% (2/7) of those without breast cancer, respectively (Cragun, 2020). In our study 57% (20/35) of moderate risk mutation carriers with breast cancer and 13% (7/55) of unaffected moderate risk mutation carriers underwent RRM. The rate of RRM in our study for women with breast cancer exceeded rate of RRM in other recently published studies, which reported that approximately one in four women diagnosed with breast cancer who underwent multigene panel testing elected for RRM; however, these other studies included women with mutations in genes that are not associated with breast cancer which may contribute to the lower rate of observed RRMs (Elsayegh, 2018; Murphy, 2020). Our study was also able to observe a strong effect of family history on RRM decision and an age‐breast cancer interaction effect. Also, our study model incorporated no mutation carriers as a negative control group which enabled further interpretation on how mutation status impacts RRM decision.

Some studies have demonstrated a survival benefit for contralateral RRM in women with *BRCA1/2*‐associated breast cancer (Evans et al., 2013; Heemskerk‐Gerritsen et al., 2015; Metcalfe et al., 2014). However, a recent study conducted by Wang et al found that breast conserving surgery for women with *BRCA*‐associated breast cancers was associated with an increase in local recurrence but had no impact on survival (Wang et al., 2022). Optimal surgical management for *BRCA* mutation carriers with breast cancer remains controversial. Additionally, currently no data exists that supports a survival benefit for breast cancer patients with moderate‐penetrance mutation carriers who elect for contralateral RRM. Given the differences in risk for a second primary cancer between these two groups, as well as the lack of data to support a survival benefit of RRM in moderate penetrance gene carriers, the preoperative counseling for these two groups is distinct. It should be noted that studies unselected for mutation status have shown no survival benefit of mastectomy versus breast conservation surgery for early‐stage breast cancer (Murphy & Gandhi, 2021). Data documenting survival benefit after RRM is limited for *BRCA* carriers and absent for healthy moderate‐penetrance gene mutation carriers and for women without known mutations (Heemskerk‐Gerritsen et al., 2019; Honold, 2018; Li, 2016). Clinicians should inform patients with moderate risk genes that data is largely insufficient to recommend RRM and explore perceived risks that may be guiding behavior. The option of RRM should be made only after careful counseling and informed risk–benefit considerations.

Weitzel et al have already demonstrated that mutations in *PALB2* and *CHEK2* are associated with having two or more breast cancers, but the same study found that neither *ATM* nor *NBN* reached statistical significance for two or more breast cancers (Weitzel, 2021). In the future, high quality longitudinal studies of moderate risk mutation carriers eventually will yield data about second primary breast cancer risk. Also, education of oncology providers is critical, as some may not fully understand genetic test results and patient decision‐making could be impacted as a result. A study of 3672 subjects who underwent genetic testing revealed that up to half of surgeons did not recognize the distinction between established pathogenic mutations and variants of uncertain significance (Kurian, 2017). Genetic counseling is encouraged for mutation carriers, when possible, for a thorough discussion about risk and preventive options (Connors, 2014; NCCN Clinical Practice Guidelines, 2020; Padamsee, 2017). While undergoing multigene panel testing may increase uncertainty for some patients in the short term, patient reported outcomes suggest that proper counseling does not increase patients' intentions for prophylactic mastectomies and may diminish uncertainty over time (Bradbury, 2020).

Family history has long been established as a factor in surgical decision (Metcalfe, 2008). Our study also corroborated research conducted by Henry et al which also showed RRM decision for mutation carriers is significantly associated with having a first degree relative with breast cancer (Henry, 2019). Women with an affected first degree relative in our study were more than three times as likely to undergo an RRM. This may partially be due to magnified risk perception; however, this study is not capable of attributing whether decisions were based on elevated perceived risk in addition to actual risk.

The univariate analysis showed that women with partners were more likely to undergo RRM. In the multivariable model, having a partner was associated with a higher likelihood of undergoing RRM, but the finding was not statistically significant. Having a partner purportedly has a greater influence on RRM decision making for women affected with breast cancer than those not facing a diagnosis due to the support a partner provides during the diagnosis process (Napoli, 2020). Additionally, cosmetic outcome may be an important factor in decision making, as this factor could be even more important in single women (Nold, 2000). It is, however, crucial to consider support networks of patients both with and without cancer since no studies to date have indicated that partner status affects the rates of RRMs among unaffected, high‐risk women. More research is needed among women with hereditary risk to better understand the role of having a partner in surgical decision making.

Age has been contended as a decisional influencer for RRM for women with breast cancer (Bhat, 2017; Chagpar, 2006; Elsayegh, 2018; Hoskins, 2012; Rodby, 2016). Literature also suggests that age factors into decision making for unaffected women. For example, younger *BRCA* carriers who are unaffected with breast cancer are more likely to undergo RRM than unaffected older women (Evans, 2009, 2019; Hoskins, 2012; Metcalfe, 2019). Evans et al demonstrated that 211 unaffected *BRCA* carriers and 3515 unaffected non mutation carriers whose remaining lifetime risk for breast cancer was 25% or higher had a reduced likelihood of undergoing RRM after age 45 (Evans, 2019). Our study did not find an association between age and RRM decision for unaffected women. The observed interaction effect of breast cancer diagnosis and age may imply that a woman's perceived future risk of breast cancer plays a larger role in decision‐making when they are facing a current breast cancer diagnosis and are already planning curative‐intent surgery. Additionally, younger women faced with a breast cancer diagnosis may be steered towards bilateral mastectomy by their surgeons more than their older counterparts, especially because younger women often tolerate surgery well, recover quickly, and have a higher remaining lifetime risk of contralateral breast cancer.

Our study found a much lower rate of RRM among Asian, Hispanic, and other patients, in comparison to non‐Hispanic White patients. Literature supports that non‐Hispanic White patients are more likely to undergo bilateral mastectomy than breast conservation surgery, and rates of RRM may therefore be higher for this study population than for others (Bradbury, 2020; Elsayegh, 2018).

### 4.1 Limitations

This was a retrospective study investigating patients receiving genetic counseling at one academic hospital in an urban setting. Women located in urban areas are more likely to undergo RRM (OR = 2.22) and the rate of RRM also differs by country (Metcalfe, 2019). The patient population constituted majority non‐Hispanic, White women and therefore underrepresents the general population demographics of the region. Our study did not capture all factors that may contribute to a woman's surgical decision‐making process. This is a complex process and previous studies have shown that many factors such as cancer worry, insurance coverage, education, income, and parity play a role (Gu, 2018; Henry, 2019; Lazow, 2019). RRM uptake has also been associated with the death of a sister with breast cancer <50 or mother <60, having children, and non‐malignant breast biopsy (Evans et al., 2021). Furthermore, the decision to undergo a RRM may be independent of mutation status and other factors. Our study was not designed to analyze how surgeon recommendation or desired cosmetic outcome impacted surgical decision. Although eligible subjects with breast cancer knew the results of their genetic test report prior to their surgery, the time between diagnosis and genetic test report was not captured and some women may have known their genetic status before their diagnosis. Additionally, the chart abstraction reflected only one time point, and the study was not designed to study surgery decisions with long‐term follow‐up.

Recent publications have challenged the classification of both *NBN* and *PALB2* as moderate penetrance genes. Two large population‐based, case–control studies published in early 2021 did not find an association between germline *NBN* mutations and breast cancer risk, in contrast, the odds ratios reported for *PALB2* mutations ranged from 3.83 to 8.04, depending on family history of breast cancer (Breast Cancer Association Consortium, 2021; Hu, 2021). Clinical recommendations for *PALB2* carriers have shifted from “RRM: Evidence insufficient, manage based on family history” in 2019 to “RRM: discuss option of risk‐reducing mastectomy” in 2020. The categorization of these genes in the moderate‐penetrance gene category aligns with the guidelines for surgical management for carriers of pathogenic variants in these genes during the study period, which was based on the understanding of the risk conferred by these genes at that time. When the study patients were tested and counseled, evidence for RRM in *PALB2* and *NBN* was considered insufficient, and NCCN guidelines stated that providers should manage carriers of mutations in these genes based on family history. Given that the surgical recommendations for *PALB2* and *NBN* carriers matched those of *ATM* and *CHEK2* carriers, patients carrying pathogenic variants in these four genes populated the “moderate penetrance” group.

## 5 CONCLUSION

This retrospective study evaluated several potential factors associated with a patient's decision to undergo RRM including mutation status, first degree relative with breast cancer, partnered status, age, and personal breast cancer diagnosis. The multivariable model revealed that these factors impact a woman's odds of electing RRM, although partnered status was not statistically significant. This study was unique in that it compared rates of RRM for moderate risk mutation carriers to “no mutation” and high‐risk mutation carriers, and the results suggest that carriers of moderate risk mutations commonly elect for RRM even without clear guidelines to support this approach. Surgical decisions are multifactorial, but providers should aim to identify the root causes of a decision for RRM in a moderate risk carrier by identifying and addressing any knowledge gaps. This study underscores the importance of educating care providers and patients on moderate risk mutations and providing access to genetic counseling wherever feasible, so that patients can be educated about genetic risk and empowered to make informed decisions about RRM.

## AUTHOR CONTRIBUTION

This study would not have been possible without all the authors listed. Each author provided support with study concept, data abstraction, analysis, and write‐up.

## FUNDING INFORMATION

The Neuroscience‐based Interventions for Cancer Risk Behavior Change NIH Grant R35CA197461 and NCI award P30CA014089 provided support for this research.

## CONFLICT OF INTEREST

The authors declare that there are no competing interests.

## ETHICS STATEMENT

This study was granted exemption by the Samuel Oschin Comprehensive Cancer Institute Protocol Review and Monitoring Committee on 5/30/2019. It was granted IRB approval by the Cedars‐Sinai Office of Research Compliance and Quality Improvement on 6/3/2019 under title name STUDY00000014: IIT2019_MCARTHUR_CHEK. Patients were not consented to this study given that it was a retrospective review study. Documentation of approval is available by request.

## Supporting information


**Appendix S1** Supporting information

## Data Availability

The data that support the findings of this study are available on request from the corresponding author. The data are not publicly available due to privacy or ethical restrictions.
