# Supplementary material for: Risk‐reducing mastectomy decisions among women with mutations in high‐ and moderate‐ penetrance breast cancer susceptibility genes
Source: Mol Genet Genomic Med. 2022 Aug 25;10(10):e2031. doi: 10.1002/mgg3.2031 (PMC9544212; doi:10.1002/mgg3.2031)
Supplement: Supplementary file 1 — Appendix S1 Supporting information [file MGG3-10-e2031-s001.docx]

^a^ Includes married and unmarried couples

^a^ Risk Reducing Mastectomy

^b^ Includes women with mutations in *ATM* (NM_000051.4)*, CHEK2_*(NM 001005735.2)*, NBN* (NM_001024688.3)*,* and *PALB2* (NM_024675.4)
